# A Review on Grafting of Biofibers for Biocomposites

**DOI:** 10.3390/ma9040303

**Published:** 2016-04-22

**Authors:** Liqing Wei, Armando G. McDonald

**Affiliations:** Department of Forest, Rangeland and Fire Sciences, University of Idaho, Moscow, ID 83844-1132, USA; liqingwei1325@gmail.com

**Keywords:** biofibers, biocomposites, bioplastics, grafting modification, cellulose, lignin, nanocellulose

## Abstract

A recent increase in the use of biofibers as low-cost and renewable reinforcement for the polymer biocomposites has been seen globally. Biofibers are classified into: lignocellulosic fibers (*i.e.*, cellulose, wood and natural fibers), nanocellulose (*i.e.*, cellulose nanocrystals and cellulose nanofibrils), and bacterial cellulose, while polymer matrix materials can be petroleum based or bio-based. Green biocomposites can be produced using both biobased fibers and polymers. Incompatibility between the hydrophilic biofibers and hydrophobic polymer matrix can cause performance failure of resulting biocomposites. Diverse efforts have focused on the modification of biofibers in order to improve the performances of biocomposites. “Grafting” copolymerization strategy can render the advantages of biofiber and impart polymer properties onto it and the performance of biocomposites can be tuned through changing grafting parameters. This review presents a short overview of various “grafting” methods which can be directly or potentially employed to enhance the interaction between biofibers and a polymer matrix for biocomposites. Major grafting techniques, including ring opening polymerization, grafting via coupling agent and free radical induced grafting, have been discussed. Improved properties such as mechanical, thermal, and water resistance have provided grafted biocomposites with new opportunities for applications in specific industries.

## 1. Introduction

A composite is usually defined as the combination of two or more different components, in which one plays the role of filler or reinforcement (such as biofibers), while the other performs as a resin or polymer matrix material. One advantage of biofibers-based composites is their low density yet higher mechanical strength and stiffness compared to glass fibers, lower impact on the environment, lower manufacturing cost, and great biodegradability. To date, biofibers-filled petroleum derived thermoplastics and thermosetting polymers have been extensively used for composites manufacturing [1,2,3,4,5,6,7]. Both the polymer matrix and reinforcement determine the overall physicochemical properties of the resulting composites.

**Figure 1:**
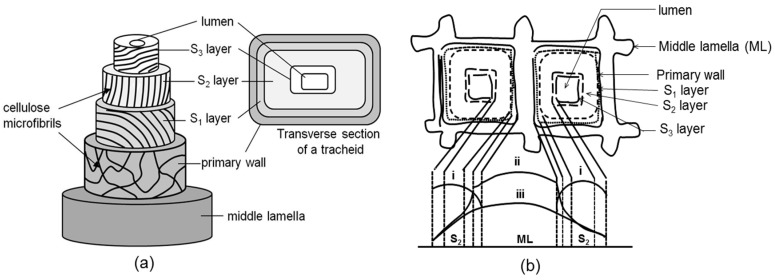
(**a**) Three-dimensional structure of the secondary cell wall of a xylem cell and (**b**) the relative amounts of cellulose, hemicellulose, and lignin across a cross-section of two wood cells (i: cellulose; ii: lignin; and iii: hemicelluloses).

**Figure 2:**
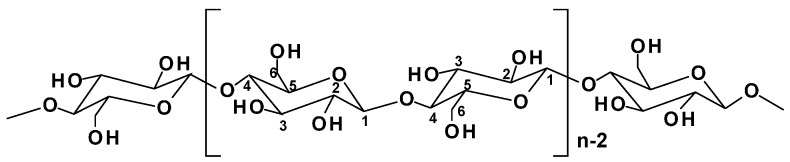
The molecular structure of cellulose (*n* = DP; the carbon positions of the ring structure are marked).

According to market data reported by Bioplastic Magazine, 352,000 tons of the wood—(74% of the market share) and natural fibers (26% of the market share) based composites were produced in 2012 in the European Union, and the wood plastic composites (WPC) will increase to 350,000 tons by 2015 [8]. About 38% of WPC was used for the automotive industry in 2012, primarily for rear shelves and trims for trunks and spare wheels, as well as in interior trims for doors; while natural fibers-based composites are mainly used for interior trims for high value doors and dashboards. Asia-Pacific is estimated to be the largest biocomposites market in the coming years due to the increasing concerns of environment and the market of construction, manufacturing and automotive sectors [9]. Although biofibers are good candidates for composites production, they also bring about challenges. The high hydrophilicity due to a large number of hydroxyl groups (Figure 1 and Figure 2) on fiber surfaces, and this can result in poor interfacial bonding/adhesion between fibers and the, generally hydrophobic, polymer matrix. Poor adhesion will make the composite vulnerable to environmental attacks (weathering, water absorption, and biodeterioration), mechanical failure, and therefore reduce service time. Composite properties can be improved via exchanging biofibers with cellulose because of its improved thermophysical properties. Such biocomposites will have great potential to be used in the automobile industry with increasing demands for recyclable new cars [10].

To reduce the hydrophilicity of cellulose fiber surface this can be achieved either through a physical or chemical treatment, and therefore to improve the interfacial adhesion between the two phases. Although these modifications result in a decrease in moisture absorption and an increase in mechanical properties, biodurability and weatherability, the processes used for cellulose modification are costly and involve toxic chemicals which could be a deterrent to its use [11].

Very recently, through the method, “grafting copolymerization”, modifying the properties of biofibers by imparting the desired and targeted properties of a polymer has attracted great interest. This method can significantly improve the stress transfer between the two phases. Review articles were published on cellulose fiber grafted to polymers via ring opening polymerization (ROP) [11,12]. However, inadequate information is available in the existing literature which summarizes other types of biofibers as well as other grafting techniques for biocomposites production.

This review intends to show the feasibilities of employing recently developed copolymerization via grafting of various biofibers as reinforcement to polymer matrices for biocomposites applications. The overall characteristics of biofibers and polymer matrix materials used in biocomposites and different modification methods to improve the biocomposites properties will be reviewed. Different grafting copolymerization techniques based on grafting reaction mechanisms are focused upon. The overall comparison of performance changes, structure manipulation, and potential applications due to grafting is discussed in detail. Finally, the review will conclude with future trends of “grafting” used for applications in biocomposites.

## 2. Biofibers

### 2.1. Lignocellulosic Biofibers

Lignocellulosic fibers (or natural fibers) are used as reinforcement in biocomposites and have been recently reviewed [2,13]. Fibers were categorized into six types: bast fibers (jute, flax, hemp, kenaf and ramie), leaf fibers (abaca, sisal and pineapple), seed fibers (coir, cotton and kapok), core fibers (kenaf, hemp and jute), grass and reed fibers (wheat, corn and rice) and other types (wood, food/agricultural residues, recycled newspaper/magazine/office paper fibers, bamboo/rattan, and roots). Natural fibers have a hollow structure, providing insulation against noise and heat. Each fibril has a complex and layered structure as shown in Figure 1 [14]. The middle lamella performs like a glue between two adjacent cells within the plant tissue. The primary wall is the first layer deposited during the formation of cell. Three layers, the outer layer (S1), middle layer (S2), and inner layer (S3), compose the secondary cell wall [15]. The distribution of chemical compositions (cellulose, hemicelluloses, and lignin) across middle lamella and wood cell walls of hardwood is shown Figure 1b [15].

In the primary wall, the orientation of cellulose microfibrils is random from 0° to 90° relative to long axial direction of the cell. For wood fibers, the angle (microfibrillar angle) between microfibrils direction and the long axis is between 50° and 89° for S1 layer [16]. These microfibrils have a diameter in the range of 10–30 nm and are typically made up of 30–100 cellulose molecules in an extended chain conformation, which provides the mechanical strength of the fibers [14]. In addition, the mechanical properties of natural fibers are also influenced by their moisture content and voids between fibers [13]. The properties of natural fiber, such as the fiber morphology, defects, crystallinity and structures, have great impacts if fibers are used as a reinforcement of the polymer matrix for composite materials.

The chemical composition of lignocellulosic biofibers varied with source and preparation methods. However, the main components of natural fibers generally include cellulose, hemicellulose, lignin, and a small amount of pectin and wax [17]. The combined effect of these components determines the overall thermal and mechanical properties of fibers. The major chemical components of selected natural fibers are summarized in Table 1.

#### 2.1.1. Cellulose

**Figure 3:**
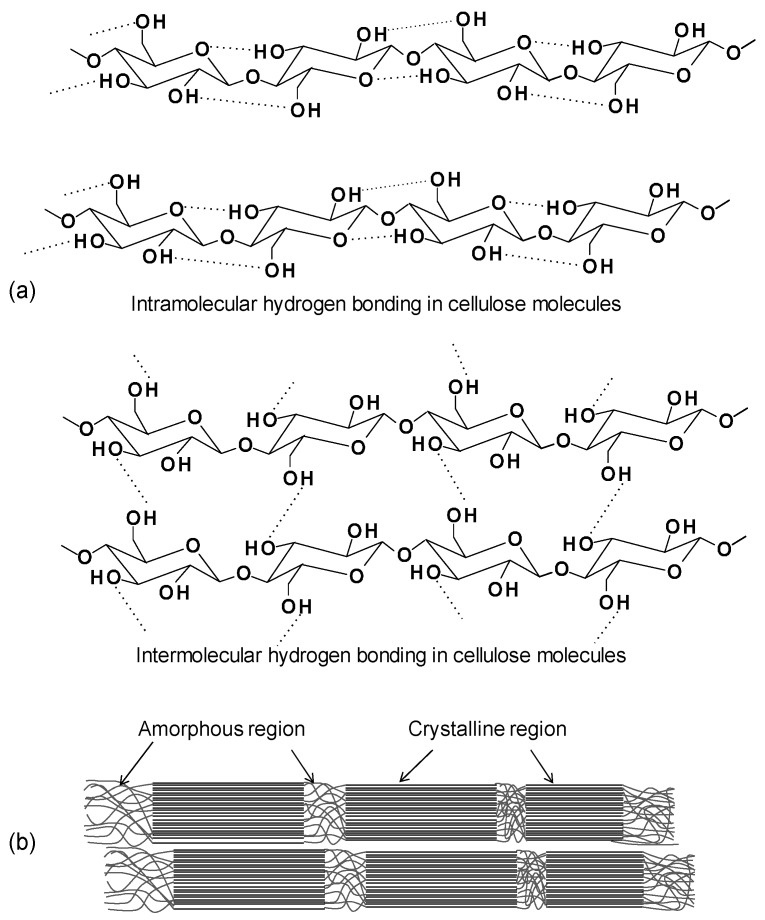
(**a**) Intra- and inter-molecular hydrogen bonding and (**b**) amorphous and crystalline region in cellulose molecules [11].

All the plant-based fibers contain a high proportion of cellulose (40%–90%) (Figure 2) [24]. Cellulose is a hydrophilic polysaccharide that consists of a linear macromolecular chain of 1 → 4 linked β-d-glucopyranosyl units to form linear chains in the cell wall [25]. All three hydroxyl groups in each monomeric unit are in the equatorial plane. The numerous hydroxyl groups on the glucose ring contribute to extensive intra- and inter-molecular C-H···O hydrogen bonds forming crystalline cellulose microfibrils (Figure 3a) [11,25,26]. The hydrogen bonding within and between cellulose chains are responsible for its high strength, stiffness, crystallinity, durability, and biocompatibility [12,26]. There are six polymorphic forms of cellulose identified, including cellulose I, II, III_I_, III_II_, IV_I_ and IV_II_. Among them, the cellulose I is found in nature and has two suballomorphs, termed I_α_ and I_β_. For both I_α_ and I_β_, cellulose chains have “parallel-up” configurations, but different hydrogen bonding patterns and therefore different crystalline structure. It was reported by Nishiyama *et al.* [27,28], that I_α_ is comprised of alternating glucose units in each chain with slightly different conformation but all molecular chains are the same, while I_β_ has two types of parallel chains. The authors used Fourier-difference analysis to demonstrate that hydrogen bonding within the I_β_ allomorph is complex and disordered.

The degree of polymerization (DP) for cellulose is about 8000 in the primary cell wall while the DP in the secondary wall is >15,000 [29]. Microfibrils are formed from bundles of cellulose chains and form larger fibrils. Cellulose microfibrils have both crystalline (highly ordered) and amorphous (less ordered) regions (Figure 3b). Generally, wood cellulose is about 60%–70% crystalline [15]. The cellulose amorphous region can absorb water more easily due to its greater accessibility. Hence, the crystallinity of cellulose is one of the key characteristics affecting its final properties, including its reactivity, mechanical performances, water absorption, and biodurability.

Cellulose is reactive due to the presence of hydroxyl groups and hence can be chemically modified to form cellulose derivatives such as cellulose esters and ethers (e.g., cellulose acetate/propionate/butyrate, methyl/ethyl cellulose, and hydroxypropyl cellulose). The reactivity of cellulose also depends on the positions of hydroxyl groups, for example, during esterification, the OH groups on the C_6_ position is 10 times more reactive than those attached on C_2_ and C_3_ [11]. The overall reactivity of the three types of OH groups are OH-C_6_ >> OH-C_2_ > OH-C_3_ [10]. The properties of cellulose derivatives are altered to native cellulose and also determined by the degree of substitutions.

#### 2.1.2. Nanocellulose

Cellulose nanomaterials have been known since the 1950s and have gained extraordinarily renewed attention recently. Generally, there are two forms of nanocellulose, the cellulose nanocrystals (CNCs) and cellulose nanofibrils (CNFs). The CNCs are also known as cellulose whiskers, nanowhiskers, or nanorods. The CNCs can be often obtained by a controlled acid hydrolysis in which the amorphous or crystalline regions of the cellulose, hemicellulose and lignin are removed. Thus, the rod-like acid-resistant CNCs are produced with reduced DP as compared to the native cellulose, and the degree of drop-off depends on the cellulose origins (e.g., cotton, ramie fibers, algae, bleached wood pulp, bacterial cellulose, or tunicate) [30,31,32]. The dimensions of CNCs vary upon the cellulose origins as well and range from 3 to 20 nm in diameter and 100 nm to several micrometers (from celluloses of tunicates, algae, bacteria) in length (aspect ratio, length/diameter, was from 10 to 100) [26,33,34,35,36]; CNCs from animal tunicate have a large aspect ratio close to 100 [37]. The Young’s modulus of CNCs can reach to the range of 120 to 170 GPa [33,37,38,39,40]. CNCs have a high specific surface area (150–170 m^2^/g) and high crystallinity (54%–90%) with reactive surfaces due to hydroxyl groups [33,41,42].

CNFs are string-like particles which can be produced via different methods, such as TEMPO-mediated oxidation (2,2,6,6,-tetrame thylpipelidine-1-oxyl radical), mechanical fibrillation using high-pressure homogenization, ultrasonic methods, and enzymatic hydrolysis [30,43]. CNFs have a high aspect ratio (4–20 nm in diameter and several micrometers in length). The CNFs generated by TEMPO oxidation have the dimensions of 3–10 nm in diameter and a few microns in length [44]. CNFs contain both crystalline and amorphous regions.

#### 2.1.3. Lignin

**Figure 4:**
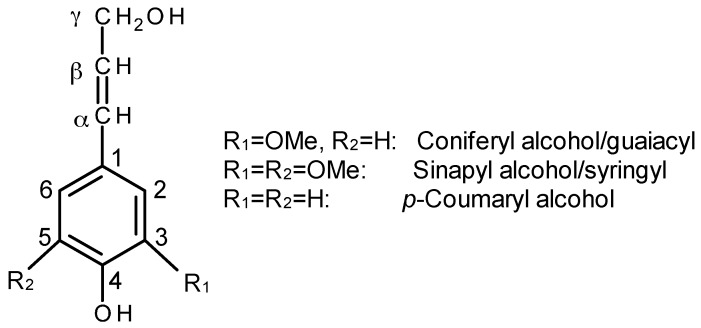
The three basic building blocks of lignin.

In addition to cellulose, lignocellulosic fibers also contain hemicellulose (branched polysaccharide) and lignin. Lignin is a complex three dimensional hydrocarbon polymer with both aliphatic and aromatic constituents. Lignin is biosynthesized from three major monomeric units, coniferyl alcohol, synapyl alcohol, and p-coumaryl alcohol (Figure 4). The composition of monolignols is dependent on plant classes and species (*i.e.*, softwood, hardwood, grass). The empirical formula for lignin is C_9_H_10_O_2_ (OCH_3_)_n_, with n being the ratio of MeO to C_9_ groups: *n* = 1.4, 0.94 and 1.18 for hardwood, softwood and grasses, respectively. The main functional groups are phenolic hydroxyl (ArOH), aliphatic hydroxyl (AlOH), carboxylic acid (COOH), and methoxy (OMe) in hardwood kraft lignin with corresponding concentration of 4.3, 1.7, 0.5, and 580 mmol/g, respectively [45]. Lignin is usually a waste byproduct of the cellulosic bioethanol and pulp and paper industry with low cost material. Hence, it has become a potential candidate as renewable materials used for sustainable polymer biocomposite as a matrix, coupling agent, coatings, and reinforcement in resin, fibers and aerogels [46,47,48,49,50,51,52,53,54,55]. With an increasing awareness of building a sustainable society, the incorporation of lignin into a biopolymer matrix as the reinforcement for biocomposites has attracted more interest. Recently, the feasibility of this idea was reviewed elsewhere [56,57].

### 2.2. Bacterial Cellulose

The species of bacteria which produces cellulose is generally called *Acetobacter xylinum*. The microbial or bacterial cellulose (BC) shows great mechanical properties due to its crystalline structure. BC has triclinic (I_α_) form crystalline structure, while wood and other higher plant cell wall celluloses are majorly comprised of monoclinic (I_β_) polymorph. In addition, BC has a higher degree of crystallinity (60%–90%) than plant cellulose and forms characteristic ribbon-like microfibrils, which gives BC excellent mechanical strength. The Young’s modulus of BCs was reported to be 15 to 35 GPa isotropically across the surface of a plane [26,58], while that of the bacterial nanocellulose (BNC) fibers can reach to 138 GPa [59]. The tensile strength of BCs can reach 450 MPa [60]. The length ofBNCs ranges from 100 nm to several mm and the diameter from 5 nm to 50 nm. BNCs also have a higher purity than other cellulose derived from natural fibers, because they do not contain hemicelluloses and lignin. In addition, the thermal expansion coefficient of BCs in the axial direction is small. Due to these mentioned fascinating characteristics of BC, it has been found to be effective for various applications such as films prepared from gel and reinforcing pulp papers or optically transparent plastics.

### 2.3. Biofibers from Other Sources

Another important biofiber source is chitin which can be found in the shells of crustacean (e.g., crab and shrimp, cuticles of insects, and cell walls of some fungi and microorganisms). Chitin has a linear structure composed of β-(1-4)-linked 2-deoxy-2-acetamido-d-glucose units (Glu-NHCOCH_3_) [61,62,63]. After chitin is deacetylated with a degree of deacetylation over 75% the so called chitosan (Glu-NH_2_) is obtained. Chitosan has excellent biodegradability and biocompatibility, hence it is being applied for biomedical uses such as kidney membrane, artificial skin, and drug delivery systems. Similar to cellulose, chemical modifications of both chitin and chitosan are of interest without changing their backbone structure and physicochemical properties. Diverse derivatives can be prepared as well [1,8,64,65,66]. Chitin nanofibers were prepared and with their modifications and applications were reported [67]. Chitin-protein composite nanofibers were recently studied by Mushi *et al.* [68].

## 3. Biocomposites

By simply blending the above mentioned biofibers with polymeric matrix, the so-called biocomposites is produced. Generally, the polymer matrix material can be conventional thermoplastics/thermosets or bio-based polymer/resins.

### 3.1. Matrix Materials

#### 3.1.1. Petroleum Based Polymer Matrix

Petrochemical based thermoplastics and thermoset matrices were extensively used for biocomposites production. These conventional thermoplastic matrix materials include polyethylene (PE), polypropylene (PP), polystyrene (PS), poly(ethylene terephthalate) (PET), and polyvinyl chloride (PVC), *etc.*, while some epoxy resin, phenol formaldehyde, and vinyl esters were used as thermoset matrices.

#### 3.1.2. Bio-Based Polymer Matrix

Driven by efforts to develop more environmentally friendly products, there is a need to replace the conventional polymer matrix materials with bioplastics for composites. Bioplastics matrix material may be bio-based polymers readily derived from renewable feed-stocks in large quantities. They are low cost and biodegradable so that a polymer can return to nature at the end of its life. Some of the most commonly known bio-based plastics in today’s market place are poly(ethylene terephthalate) (PET), polyethylene (PE), polypropylene (PP), polyamides (PA), poly(trimethylene terephthalate) (PTT), poly(lactic acid) (PLA), polyhydroxyalkanoates (PHAs), poly(propylene carbonate) (PPC), thermoplastic starch (TPS), soy protein based resins, and other biodegradable polyesters [*i.e.*, poly(caprolactone) (PCL), poly(butylene succinate) (PBS) and poly(butylene succinate-co-adipate) (PBSA)] [69,70,71,72,73,74,75,76]. Except for bio-based PE, PP, PTT, PET, and PA, all other bio-based polymers just mentioned are biodegradable at different levels. Among all of the bioplastics mentioned above, the microbial PHA, PLA, biodegradable starch-based blends, PBS/PBSA, PCL, PPC, and soy protein-based resin have gained significantly in interest since they could be completely biodegradable in the appropriate environments. In addition, apart from their good biodegradability, PHAs and PLAs can be biosynthesized from renewable resources which allows large scale production [64,69,70,72,74].

**Figure 5:**
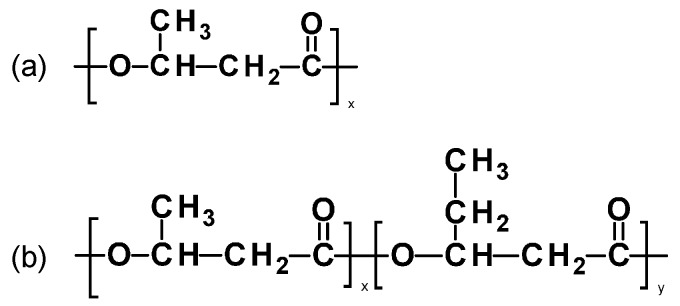
Chemical structures of (**a**) poly(β-hydroxybutyrate) (PHB) and (**b**) poly(3-hydroxybutyrate-co-3-hydroxyvalerate (PHBV).

Polyhydroxyalkanoate (PHA) is a special group of polyesters produced by a wide variety of microorganisms as an internal carbon and energy reservoir as part of their survival medium. All metabolized carbon sources can be used for the production of PHA such as fatty acids and carbohydrate [77]. A large amount of literature is available to describe the biosynthesis [78,79,80,81,82,83,84], characterization of the PHA properties [72,85,86,87,88], opportunities and challenges of PHA products on the global market [89], and applications of PHA [77]. Poly(β-hydroxybutyrate) (PHB), Figure 5a, is a homopolymer of 3-hydroxybutyrate and was the first discovered member of the PHA family in 1925 by Lemoigne, while its copolymer the poly(3-hydroxybutyrate-co-3-hydroxyvalerate) (PHBV, Figure 5b) has attracted great attention with improved thermomechanical properties as compared to PHB.

**Figure 6:**
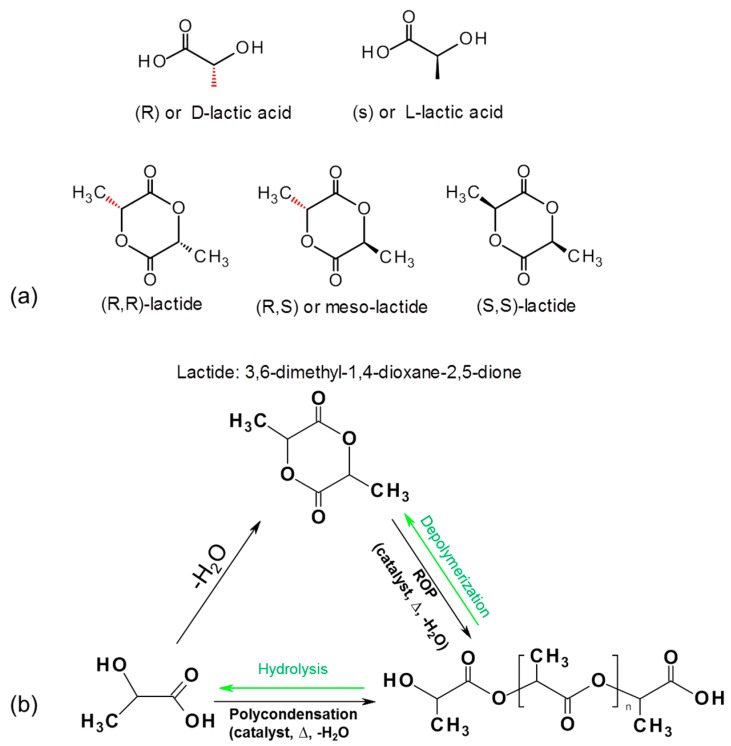
Stereochemistry of the lactic acid (LA) and lactide monomers (**a**) and chemistry of the interconversion between LA, lactide and poly(lactic acid) (PLA) (**b**). Note: Reversible depolymerization and hydrolysis steps are highlight in green color [94].

Polylactic acid (PLA) is a synthetically produced biodegradable plastic from bioderived monomers from renewable resources. PLA can be synthesized via different polymerization routes, which is shown in Figure 6. Based on the stereochemistry of lactic acid (LA) and lactide monomers (Figure 6a), several distinct forms of PLA exist: poly-l-lactic acid (PLLA) is the product resulting from polymerization of l-lactide (also known as (S,S) or l-lactide), while poly-d-lactic acid (PDLA) is synthesized from d-lactide (also known as (R,R)-lactide). Commercial PLA homopolymer is generally prepared from l-lactide (LLA) which can be derived from corn starch fermentation with relatively high enantiomeric purity [90,91]. To date, the major PLA suppliers have been Cargill (in USA known as Ingreo™, under trade name Nature Works, (Minnetonka, MN, USA), Mitsui Chemicals, Inc. (Tokyo, Japan), Purac (Gorinchem, The Netherlands), and Teijin Limited (Osaka, Japan). The increased production of PLA has facilitated an increase in its research and development activities. The number of publications related to PLA increased dramatically over the past decade, which is due to the increasing demands of sustainable and eco-efficient bioplastics. A viable end-of-life option of PLA is chemically recycling back to monomers through hydrolysis into lactic acid which will enter into the polycondensation step [92], or reversible polymerization to lactide monomer using catalytic thermal depolymerization [93]. Work is in progress to upscale the depolymerizatin of PLA back to lactide, which, if successful, it is believed will reduce the market prices of PLA significantly.

### 3.2. Green Composites

By replacing the conventional polymer matrix materials with bio-based polymers such as starch and soy protein based resins, PHAs, and PLAs, green biocomposites is achieved [17,29,95,96,97,98]. Green composites are advantageous materials as they can completely decompose after being discarded without any environmental impact at the end of life cycle. Commonly known green composites are discussed below.

#### 3.2.1. PHA Based Biocomposites

Biofibers have been incorporated into the PHA polymer matrix to improve its thermomechanical properties and reduce the cost of the final products (biocomposites) [96,99,100]. PHB/PHBV based biocomposites reinforced with bast fibers [101], straw/husk [102,103], bamboo fibers [104], distiller’s dried grains with solubles (DDGS) [105,106], and potato peel fermentation residue [107], have been reported. In these cases, the thermal stability was increased by addition of fibers while Shanks *et al.* [108] showed improvement in bending/storage modulus by addition of flax fibers. Barkoula and colleagues [109] studied properties of flax fibers-reinforced PHB composites and showed that PHB was toughened in these biocomposites. Furthermore, to the presence of fibers stimulated the initial biodegradation of these biocomposites by its moisture uptake. Srithep *et al.* [110] prepared nanocomposites with PHBV reinforced with nanofibrillated cellulose (NFC). Addition of NFC increased the tensile modulus significantly because it served as nucleation agent that facilitated PHBV crystallization. Compounding PHB with starch for transparent film was also studied by Godbole *et al.* [98]. The results revealed that blended films had a single glass transition temperature. The nature of all combinations was found to be crystalline. As the cost of PHB is high as compared to conventional plastics and because starch is abundantly available at a very low cost, blending of starch up to 30% in PHB would help to reduce the price of bioplastics, without sacrificing the physical properties. This biocomposite could be used as a coating material for food packaging materials. Coats *et al.* [111] prepared biocomposites with PHB-rich biomass compounded with pine wood fibers and compared properties with purified PHB based composites. The former composites exhibited comparable strength to the later type. This alternative approach to utilize PHA biomass without further purification of PHA polymers from bacterial cells could reduce the cost of the biocomposite.

#### 3.2.2. PLA Based Biocomposites

Similar to PHAs based biocomposites, PLA is another attractive biodegradable polymer matrix. Various properties for different biofibers, such as hemp, flax, coir, agricultural waste, starch, recycled paper fibers and wood fibers as well as cellulose/nanocellulose and lignin have been used as reinforcement to PLA matrices [65,97,104,112,113,114,115,116,117,118,119,120,121] and reviewed [2,13,42,122]. By compounding the fibers with PLA the mechanical properties were improved as compared with neat PLA. For example, quasi-static tensile modulus of the unidirectional long artichoke fibers reinforced PLA green biocomposites was increased by 40% as compared with neat PLA [120].

Arrieta *et al.* [123] prepared the PLA/PHB/CNC blends and evaluated the mechanical, barrier and degradation properties of the biocomposites film. The ternary system showed enhanced mechanical properties, improved water resistance, and reduced oxygen and UV-light transmission. The disintegration of PLA was delayed due to addition of PHB; in contrast, CNC facilitated its degradation, which suggests potential applications for packaging materials of the biocomposites.

#### 3.2.3. Other Commonly Studied Biocomposites

Another commonly studied bioplastic matrix material for biocomposites is PCL. In a recent study by Hejna *et al.* [124], agricultural waste (brewer’s spent grain (BSG) and wheat bran (WB)) were compounded with PCL for biocomposites production. The influence of BSG biofiber loadings on processing, static and dynamic mechanical properties, thermal properties was investigated. All the results indicated that BSG and WB-produced biocomposites is an avenue to effectively manage agricultural wastes and develop valuable industrial and practical applications.

Wang *et al.* [125] investigated the mechanical properties and water resistance of BC reinforced TPS/PLA biocomposites and modified with sodium hexametaphosphate (SHMP). It was found this modification approach significantly improved the tensile strength and impact strength values of composites as compared to initial BC-reinforced TPS.

#### 3.2.4. Approaches to Improve the Performance of Biocomposites by Modifications

Biofibers have promising properties as reinforcing materials for biocomposites production; however, the biggest obstacle for the practical application of biocomposites is the poor stress transfer between the reinforcement and polymer matrix. Generally, the stress transfer from matrix to filler/reinforcement is strongly dependent on the degree of bonding between the two phases [126,127]. The poor interfacial adhesion at the two interfaces means that the full capabilities of the composite cannot be exploited, resulting in the cellulose being just a filler and not a true reinforcement, that could weaken its mechanical properties, and thus reducing its life span [127]. Two major types of modifications have been conducted to facilitate the stress transfer at the interphase [128,129,130,131]: (i) reducing the hydrophobicity of biofibers; (ii) using coupling agent. The first step has been extensively employed and achieved either through a physical treatment (e.g., cold plasma treatment) [132] or chemical treatment (e.g., organosilanes, isocyanates, etherification and esterification). These modifications result in a decrease in moisture absorption and an increase in mechanical properties, biodurability and weatherability [127]. Chemical treatments of natural fiber for use in natural fiber-reinforced petroleum based polymer composites were reviewed elsewhere [133].

One of the most effective methods for chemical modification of cellulose was found to attach polymer chains directly to cellulose fiber surfaces, which was named “grafting”. Via grafting, biofibers are covalently bonded onto the polymer matrix using a wide variety of polymerization techniques. This can effectively improve the compatibility and stress transfer between the two phases of the created biocomposite. Hence, the performances of biocomposites can be improved significantly.

## 4. Grafting Modifications of Biofibers for Biocomposites

### 4.1. Grafting Techniques

To date, graft copolymerization of biofibers with polymer for biocomposites can be categorized into three groups: (i) grafting of fiber with a single monomer; (ii) grafting with a mixture of two or more monomers; and (iii) grafting with the polymer directly. Final properties of grafted composites can be tailored by changing the processing parameters during polymerization such as the nature of the biofiber/polymer backbone, monomer/oligomer types, and the effect of initiator. Different means have been investigated to copolymerize different monomers onto biofiber backbones, especially for cellulose and lignin. These techniques majorly include living polymerization, free radical initiation, ionic, photochemical, plasma induced initiation, plasma radiation induced grafting, and enzyme grafting. For example, maleic anhydride has been utilized as a grafting monomer to functionalize biopolymers and used as coupling agent to promote the interfacial adhesion of fiber/polymer interphases in biocomposites [96].

### 4.2. Grafting via Living Polymerizaiton Technique

By grafting polymers onto cellulose its stiffness can be retained and the thermoplasticity of the polymer matrix can be imparted. The applications of biocomposites are expected to be broadened accordingly. Among all the grafting techniques, ring opening polymerization (ROP), a living polymerization technique, has been mostly used to graft polymer to/from cellulose fibers, followed by radical initiation because ROP of lactide has been well established for PLA synthesis. Cellulose modification by polymer grafting via ROP has been reviewed [12,37]. This method was demonstrated to be potentially used for biocomposites applications. Apart from ROP, grafting via free radical initiation has also been applied to the grafting copolymerization process and can be used for biofibers-reinforced biocomposites.

#### 4.2.1. Ring Opening Polymerization for Biocomposites

**Figure 7:**
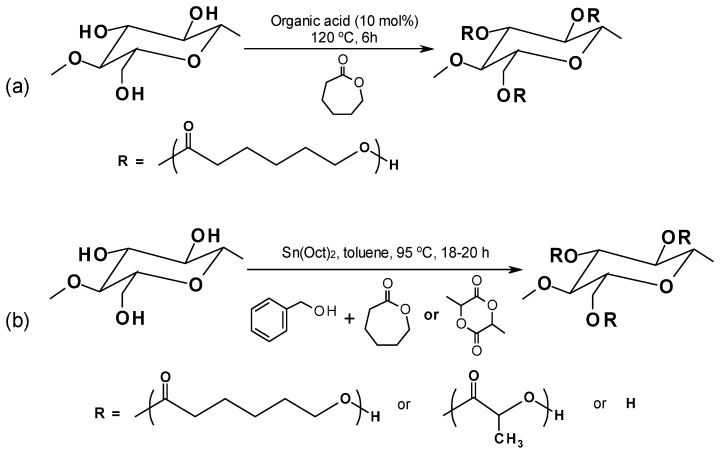
Organocatalytic ROP process of (**a**) cyclic ε-caprolactone and (**b**) cyclic ε-caprolactone or lactide with cellulose [132,133].

‎The organocatalytic ROP process leaves no metal residues in the resulting biocomposites and allows for quite good control of the final product characteristics (*i.e.*, controlled molecular weight, narrow molecular weight distribution) [134]. In Hafren and Cordova’s work [135] an organic acid was effectively used to catalyze the ROP for producing PCL-graft-cellulose directly for the first time (Figure 7a). Lönnberg *et al.* [136] successfully grafted cyclic monomers, ε-caprolactone and lactide, to the cellulose surface via covalent bonding with the respective polymers by using the Sn(Oct)_2_-catalyzed ROP process (Figure 7b). Benzyl alcohol was used as an initiator and the grafting density can be controlled by the ratio of initiator to monomers.

Outstanding mechanical properties were obtained by blending nanocellulose and polymer matrix at low filler loadings [137]. Diverse efforts have been undertaken to modify nanocellulose via grafting to improve the compatibility between the two phases. PCL-grafted-CNC bionanocomposite was prepared via Sn(Oct)_2_-catalyzed ROP mechanism [138]. Fourier Transform Infrared (FT-IR) spectroscopy, Time-of-Flight Secondary Ion Mass Spectrometry (TOF-SIMS), and X-ray diffraction (XRD) analyses were used to confirm and demonstrate the efficiency of the PCLgrafting via the ROP method onto CNCs. The thermal and mechanical properties and crystalline structures of the grafted composites were studied by differential scanning calorimeter (DSC), dynamic mechanical analysis (DMA) and tensile testing. The obtained PCL-graft-CNC showed an improved mechanical performance and the grafted composites became more ductile at room temperature. The Young’s modulus was increased by 152% for bionanocomposites reinforced with 40% PCL-graft-CNC, and elongation at break was increased by 333% for composites containing 30% of the grafted copolymer as compared to the composites filled with unmodified CNCs. It was concluded that the surface-grafted CNCs could act as nucleation sites, which facilitated the crystallization of the polymer matrix. It was evidenced that the grafting modification enhanced the compatibility of CNC/polymer matrix and thus the final properties of the grafted bionanocomposites.

Ethyl cellulose-graft-poly(ε-caprolactone)-block-poly(l-lactide) (EC-g-PCL-b-PLLA) block copolymers were prepared by sequential ROP [139]. The block length was tuned by changing the molar ratios of ε-caprolactone monomer to ethyl cellulose and the l-lactide to ε-caprolactone. Thermal properties and crystalline nature of EC-g-PCL and EC-g-PCL-b-PLLA copolymers were different from those of linear PCL. The *in vitro* degradation was facilitated with the addition of PLLA blocks in the EC-g-PCL-b-PLLA copolymers as compared to linear PCL and EC-g-PCL. The biodegradation rate can be controlled by monitoring the grafting efficiency. This can enable the grafted biocomposites to be used in the controlled release applications.

In the study by Goffin *et al.* [140], CNC was grafting modified via ROP with l-lactide to obtain the PLA-graft-CNC copolymer and then filled into PLA matrix to prepare the PLA/PLA-graft-CNC green biocomposites. Above the glass transition temperature, the stiffness of PLA-graft-CNC reinforced PLA biocomposite (PLA/PLA-graft-CNC) was increased as compared to the PLA/CNC simple blended biocomposite, which was caused by the higher crystallinity due to grafting modification of CNC [140]. With the incorporation of PLA-graft-CNC into PLA/CNC composite matrix, the final product was colorless and more transparent then the unmodified CNC filled PLA biocomposite. This suggested that the grafting modification of CNC limited their thermal degradation, allowing them to be processed at higher temperature when subjected to melt processing. This broadens the PLA/CNC composite applications into new modern applications such as 3D printing materials, packaging, and electronic industries.

Nanocomposites of nanopaper fibers were obtained via surface initiated ROP of ε-caprolactone [141]. Two types of catalysts, titanium n-butoxide (Ti(On-Bu)_4_) and tin octoate (Sn(Oct)_2_) were used. It was found that the high surface area of nanopaper fibers gave higher grafting efficiency with Sn(Oct)_2_. The mechanical properties of grafted copolymer were superior to those of pure PCL.

In addition to cellulose, to utilize the low-cost but renewable lignin fibers through compounding with biopolymers for biocomposite applications is gaining attention. However, it is difficult to blend lignin with other polymers due to poor biocompatibility. Lignin has an abundance of accessible OH groups on its surfaces, which can be modified as cellulose. Recently, Chung *et al.* [52] prepared the lignin-graft-PLA copolymer via ROP process. The copolymerization was catalyzed by triazabicyclodecene (TBD). The molecular structure of the PLA in the copolymer was controlled by manipulating the ratio of lignin/lactide monomers and the lignin pretreatment (acetylation). Results showed that the aliphatic OH groups were preferred over the phenolic OH groups during grafting. The obtained PLA-graft-lignin copolymer was subsequently used as a dispersion modifier for the PLA-lignin biocomposite and properties were studied and compared with the simple blended composites made from PLA and unmodified lignin. The glass transition temperature of PLA-graft-lignin was increased by 40 °C when lignin content was increased from 10% to 50%. The addition of PLA-graft-lignin into the biocomposite resulted in a respective increase in tensile strength and strain by 16% and 9% without diminishing the Young’s modulus. It was found the biocomposites modified with 10% PLA-graft-lignin displayed excellent UV (UVC, UVB, and UVA) resistance.

#### 4.2.2. Free Radical Grafting

Grafting of various monomers onto cellulose fibers or cellulose derivatives is an extensively used tool for cellulose modification. The grafting reaction is carried out with the aid of free radical initiators. Free radicals are created by either chemical (ceric ammonium nitrate (CAN), various persulfates, and Fenton reagent (Fe(_II_)–H_2_O_2_)), thermal (azobisisobutyronitrile (AIBN) and organic peroxide), or irradiation (UV, gamma, and electron/plasma ion beam) initiation [11]. For example, vinyl or acrylic monomers are grafted onto cellulose backbone is an important technique to modify the cellulose and give new properties of the resulting composites. Generally, three major steps are involved in the free radical initiation polymerization: (i) initiation; (ii) propagation; and (iii) termination. In step (i) the free radicals are formed on the surface of cellulose substrate; followed by (ii) and (iii) in which the monomers react with the free radical sites to propagate and terminate with new polymer chains that are covalently bonded to the cellulose backbone. Free radicals would be generated at the newly formed polymer chains and terminated with other radical sites on polymer chains, and thus branches will be formed.

Cellulosic Grewia optiva fibers were grafted with methyl acrylate monomer to form PMA-graft-cellulose copolymers [142]. The process was carried out under microwave conditions using ferrous ammonium sulfate-potassium per sulfate as redox free radical initiator. The grafting efficiency (GE) was increased with microwave power up to 70%, while a maximum at 37% GE was reached at the optimized parameters (microwave radiation power, ratio of monomer, solvent and initiator concentrations). The grafted copolymers showed an increase in chemical resistance thermal stability. This provides a proficient method to broaden the applications of biofibers for use in biocomposites. A similar method was applied for synthesizing poly(butyl acrylate)-graft-cellulose copolymers [143]. The graft copolymers have been found to be more resistant to moisture and also showed better resistance to chemical and thermal deterioration.

Gum rosin polymer-graft-lignin composites were prepared via atom transfer radical polymerization (ATRP) [144]. The 2-bromoisobutyryl ester-modified lignin was produced and used as a macroinitiator. Three different monomers derived from dehydroabietic acid (DA) were separately attached to lignin through an esterification process. Grafting of both DA and rosin polymers significantly enhanced the hydrophobicity of lignin. Water uptake of the grafted composite was significantly reduced. The ATRP technique has also been used to synthesize CNC grafted to poly(acrylic acid) (PAA) materials with a brush type structure with controlled chain length using a Cu-mediated surface initiated controlled radical polymerization [145]. The CNCs were first modified to create an initiator moiety on the surface. This new modification route for CNCs enables further advanced uses for functionalized CNC and applications.

Zoppe *et al.* [146] prepared CNC-graft-poly(*N*-isopropylacrylamide) biocomposite brushes via surface-initiated single-electron transfer living radical polymerization (SI-SET-LRP). The CNCs were produced from sulfuric acid hydrolyzed ramie fibers (3–15 nm × 50–250 nm). The grafting was confirmed by FTIR and X-ray photoelectron spectroscopies. It was expected that the interfacial interactions between CNCs and the polymer matrix can be controlled by changing the reaction temperature and monomer loading. Their results provided insight for the development of temperature-responsive materials from CNCs.

In order to make the CNC compatible with natural rubber, Parambath Kanoth *et al.* prepared biocomposites using the free radical initiation technique [147]. Firstly, the cellulose was functionalized with mercaptoundecanoic acid to get mercaptoundecanoyl CNC bearing thiol groups, and then the photochemically initiated cross-linking reaction occurs between the double bonds of natural rubber and thiol groups of CNC. This technique improved the interfacial bonding between CNC and the rubber matrix and thus improved their thermomechanical properties. For example, the tensile strength and elongation to break were increased 2.4 and 1.6 fold, respectively, upon modification as compared to blends.

Additionally, cellulose can be treated with high energy radiation (e.g., γ-rays from radioactive isotopes or electron beams) to generate radicals together with chain scission [11,148]. Irradiation of cellulose would produce free radicals which further initiate the polymerization of vinyl and acrylic monomers, and thus the cellulose-graft-polymer biocomposites are produced. Sisal fibers were grafted with co-monomer of styrene and ethyl acrylate initiated by gamma irradiation [149]. The mechanical properties experienced deterioration upon irradiation regardless of the irradiation atmosphere, while dye uptake for basic and direct dyes were found to slightly increased at low graft efficiency and then to decrease with increasing grafting extent. For the disperse dye, the dye uptake was increased with increasing grafting levels.

Microwave radiation was used to graft *Luffa cylindrica* [150] and *Grewia optiva* fibers [151] to methyl acrylate/acrylamide. The grafted copolymer showed improved thermal, structural, chemical, and morphological properties and this technique can be potentially used as an adsorbent for water purification and other industrial applications. The main advantage of using microwave chemical grafting strategy is in the use of a liquid medium with a wide range of solvents that can be used, which allows better dispersion of biofibers in the polymer matrix. However, these manufacturing methods are not scalable and non-economic. Although radiation induced grafting is relatively easier to operate, it is always difficult to control the radical generation process.

**Figure 8:**
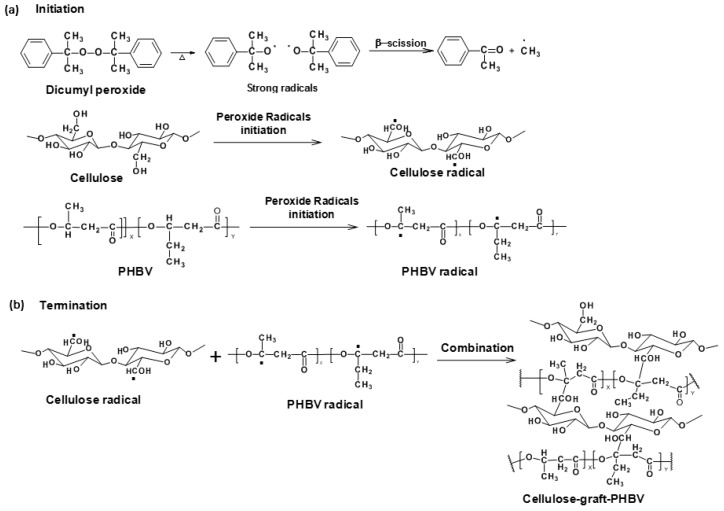
Reaction mechanism of peroxide radical initiated grafting of PHBV onto cellulose: (**a**) radical initiation and (**b**) reaction termination [149].

Melt-compounding techniques, such as extrusion and injection molding, are usually used for thermoplastic processing, which are solvent free and scalable (for blown film especially). In recent studies [152,153], cellulose and PHB/PHBV composites were manufactured via *in situ* reactive extrusion with dicumyl peroxide (DCP) free radical initiation at high temperature (Figure 8). The grafted copolymers at the interfaces of cellulose and PHB/PHBV matrix performed like an interfacial coupling agent. Grafting tended to interact with both the hydrophilic fibers and the hydrophobic PHB or PHBV matrix. The grafting mechanism was confirmed by electron spin resonance (ESR) analysis and showed the presence of radicals produced by DCP radical initiation for the first time. The grafted copolymer structure was determined by nuclear magnetic resonance spectroscopy. The biocomposites were also characterized by scanning electron microscopy and dynamic mechanical analysis and indicated good interfacial bonding and compatibility between the two phases. The mechanical properties of the biocomposites were improved by grafting due to improved stress transfer between the two interphases of the cellulose fiber/polymer matrix as compared to the blend control composite [152]. The better mechanical performances of grafted biocomposites also contributed to the crosslinks formed among the PHB or PHBV polymer chains. The crystallinity of PHB, PHBV and cellulose in the grafted biocomposite were reduced as determined by FTIR spectroscopy, X-ray diffraction, and DSC analyses. The reduction of crystallinity indicated that the grafting reaction occurred not just in the amorphous region of cellulose fibers but also slightly in crystalline domains. Reduced crystal sizes suggested that the brittleness of PHB was decreased due to grafting; in other words, flexibility of PHB was introduced via grafting. Thermogravimetric analysis showed that the grafted copolymer was stabilized relative to PHB. By varying the reaction parameters, the compositions (%PHB and %cellulose) of resultant cellulose-graft-PHB copolymer are expected to be manipulated to obtain tunable properties.

This *in-situ* reactive extrusion process offers an effective approach to improve the properties of biocomposite materials from sustainable resources and has also been employed to produce thermally recyclable PLA-graft-CNC biocomposites [154]. The grafted portion was isolated and re-fed into the biocomposites thermal processing as coupling agent. The presence of chemical crosslinks between the CNC and PLA matrix gave efficient modulus transfer, improving the tensile strength and Young’s modulus by 40% and 490%, respectively. This provided an alternative utilization of CNC and PLA for biodegradable and recyclable biocomposites films for packaging materials.

Kraft lignin was successfully grafted onto a high density polyethylene (HDPE) matrix by the DCP free radical initiation [155]. Maleated lignin was generated via esterification with maleic anhydride prior to the grafting reaction. Better compatibility of lignin and HDPE was observed than for the maleated lignin and HEPE composites system. Due to grafting, the thermal stability of maleated lignin and HDPE composites was improved as well. Csikós *et al.* [156] used reactive extrusion process with aid of peroxide to improve the interfacial adhesion of wood fiber and PLA biocomposites. In this study, the functionalized PLA with maleic anhydride was prepared first and then compounded and radically-initiated grafted with wood fibers for biocomposites. The functionalized PLA proved to be an efficient coupling agent in PLA/wood composites. The mechanical strength was increased due to grafting. This opens the window of utilizing low-value lignin as renewable reinforcement for biocomposites used for structural materials for automotive or construction purposes.

### 4.3. Grafting via Coupling Agent

Another approach to enhance the compatabilization between biofibers reinforcement and polymer matrix is to use a grafting or coupling agent, such as isocyanates. *N*-Octadecyl isocyanate was used as grafting agent to improve the compatibility of CNC and microfibrillated cellulose extracted from sisal fibers to PCL polymer matrix [157]. Nanocomposite films were prepared by solvent casting. The chemically grafted composites improved the compatibility between the two phases and helped dispersion of CNCs into the PCL matrix. Mechanical properties of the grafted composites were improved in terms of stiffness and ductility. Transition temperatures and crystallinity were increased with fiber content.

Methylenediphenyl diisocyanate (MDI) was used as coupling agent for biocomposites from PLA and starch [121]. The authors found the presence of MDI in the PLA/starch composite did not affect the thermal decomposition profile when the composites were stored at 25 °C and relative humidity fluctuating between 90% and 30% for up to 180 days. The mechanical properties of PLA and PLA/starch-based composites sealed in PE plastic bags were not changed during 30-days of storage in fluctuating humidity conditions (30%–90%).

Magniez *et al.* [158] found the interfacial interaction was enhanced by using poly-(ethylene glycol)-b-poly-(l-lactide) (PEG-PLLA) block copolymers as coupling agent for jute fiber and PLA biocomposites. A solvent solution of the copolymer was deposited onto the fibrous substrate. The strong interaction between the fiber-matrix interphase contributed to the intra- and inter-molecular hydrogen bonds between these two phases. By adjusting the amphiphilicity and the type of building blocks of copolymer, the surface properties of the biocomposites can be tuned.

Biocomposites from biodegradable polymers onto cellulose and cellulose derivatives have been reported [159,160]. Samain *et al.* [159] investigated the grafting of three biodegradable polymers, PLA, PCL and PHA onto cellulose paper and microcrystalline cellulose fibers. Firstly, the low molecular weight telechelic OH-terminated oligomers were prepared, followed by the graft copolymerization. Specifically, the reactive carboxylic end groups were formed for PHA by thermal treatment, while PCL oligomers were prepared via ROP. This was followed by producing oligomers with an acid chloride group at the end of PHA, PLA and PCL. These were prepared with the aid of thionyl chloride. The grafting reaction was initiated at high temperature between chloride oligomers and cellulose. In the study by Yu *et al.*, telechelic OH-terminated PHBV oligomers were prepared first by a transesterification procedure. PHBV-graft-ethyl cellulose copolymers were synthesized using 1,6-hexamethylene diisocyanate (HDI) as a grafting agent. The grafting conditions, such as the reaction time, temperature and mass ratio of PHBV oligomer to EC, were optimized to obtain the maximum grafting percentage. Compared with neat PHBV, the crystallinity of the grafted copolymer decreased and the moisture resistance was improved significantly.

Very recently, biocomposites made from PP and argan nut shell as reinforcement was studied [161]. In this work, styrene–(ethylene–butene)–styrene triblock copolymer grafted with maleic anhydride (SEBS-g-MA) was used as a coupling agent to enhance the interaction adhesion between filler and polymer matrix. SEM micrographs revealed that good filler dispersion into matrix with the aid of the coupling agent. The addition of coupling agent improved the thermal stability of PP.

In addition to these above mentioned major grafting strategies, PHBV and poly(3-hydroxyoctanoate) (PHO) have been successfully grafted onto chitosan by condensation with reaction occurred between amine groups of chitosan and carboxylic end of PHBV and PHO [61]. These grafted biocomposites could be used as new materials in medical applications, such as tissue engineering and drug delivery systems.

## 5. Conclusions

Naturally derived biofibers have been frequently used as reinforcement for polymer composite applications, especially the natural cellulose, lignin, microbial cellulose, and nanocellulose fibers, due to their low-cost and light weight. To reduce the impact on the environment, bio-based plastics are used to replace the conventional polymer matrix, giving completely green biocomposites. By incorporating various biofibers into polymer matrix, the prices of the bioplastics can be reduced and the properties can be improved. However, biofibers contain a large amount of hydroxyl groups, making them highly hydrophilic; whereas the polymer matrices materials are generally hydrophobic. The poor interfacial adhesion at the interphases limits the applications of biocomposites exposed to water, heat, and UV light. Various approaches have been employed to modify the fiber surfaces to improve the compatibility between the reinforcement and polymer matrix. On the other hand, “grafting” has been shown to be effective at imparting the desired and targeted properties of polymers onto cellulose, nanocellulose, and lignin (or their derivatives) as well as enhancing the interaction between interphases. Ring opening polymerization, coupling agent-induced grafting, and grafting via free radical initiated copolymerization techniques have been investigated. These grafting copolymerization techniques have offered an alternative modification approach to biofibers as a reinforcement of polymers for biocomposites including green composites and bionanocomposites production. This can potentially broaden the biocomposites utilized in numerous modern applications in the future, such as packaging materials, structural materials, coatings, biomedical devices, drug delivery release, electronic devices, waste-water treatment, and in the automotive industry.

## Figures and Tables

**Table 1:** Major chemical composition of selected natural fibers (%).

Type of Fiber	Cellulose	Hemicellulose	Lignin	References
Black locust	41.61	17.66	26.70	[18]
Hybrid poplar	44.70	18.55	26.44	[18]
Eucalyptus	49.50	13.07	27.71	[18]
Pine	44.55	21.90	27.67	[18]
Switchgrass	31.98	25.19	18.13	[18]
Bagasse	54.3–55.2	16.8–29.7	24.3–25.3	[19]
Bamboo	34.50	20.50	26.00	[20,21]
Rattan	35.6–52.9	22.8–34.7	21.0–22.0	[22]
Flax	70.50	16.50	2.50	[20,21]
Hemp	70–92	18-22	3–5	[23]
Kenaf	53.50	21.00	17.00	[20,21]
Jute	67.00	16.00	9.00	[20,21]
Oil palm	14.3–65.2	12.5–38.7	17.3–26.5	[22]
